# Cardoon cake as a sustainable alternative ingredient in broiler feeding

**DOI:** 10.1080/1828051X.2025.2503395

**Published:** 2025-05-23

**Authors:** Federica Mannelli, Federica Scicutella, Giuseppe Conte, Matteo Daghio, Carlo Viti, Ilaria Galigani, Elisabetta Toni, Alessandra Miele, Giovanni Brajon, Lapo Nannucci, Arianna Buccioni

**Affiliations:** a Dipartimento di Scienze e Tecnologie Agrarie, Alimentari, Ambientali e Forestali, University of Florence, Florence, Italy; b Dipartimento di Scienze Agrarie, Alimentari e Agro-ambientali, University of Pisa, Pisa, Italy; c Istituto Zooprofilattico Sperimentale del Lazio e della Toscana ‘M. Aleandri’, Florence, Italy

**Keywords:** Poultry feeding, soybean meal, cardoon meal, sustainability, gut microbiota

## Abstract

A strategy to improve poultry meat production sustainability is the use of by-products from agro-industries, such as cardoon cake (CC). Due to limited literature available, this trial aimed to study the effect of CC as an alternative protein source to soybean meal (SM) or insect meal (black soldier fly, *Hermaetia illucens*) on broilers’ performance, welfare, and meat quality. Eighty day-old chickens were allocated into 4 dietary treatments composed of 5 replicated pens (4 animals per pen): control group (C) fed a basal diet with SM as main protein source; HI15 group fed C with 15% of SM replaced with *H. illucens* meal; CC15 group fed C with 15% SM replaced with CC; CC20 group fed C with 20% of SM replaced with CC. In the starter period, chicks from CC15 showed similar trends in weight gain (WG), feed intake (FI), and feed conversion ratio (FCR) to the C group; FCR was also comparable to that in HI15, but FI was lower with respect to HI15. In the finisher period, WG in CC15 and CC20 groups did not differ from the C and HI15 groups, but the FCR worsened with respect to C whilst remaining comparable to HI15. The meat fatty acid profile from animals fed CC was rich in oleic acid. *Bifidobacterium* was detected in the CC20 group only, which also showed significantly lower relative abundance of *Bacteroides*, *Monoglobus*, UCG-009, *Colidextribacter*, and NK4A214_group, and significantly higher relative abundance of *Latilactobacillus*. These results show that CC can be used for broilers feeding.

## HIGHLIGHTS

- Cardoon cake was tested as sustainable alternative protein source to soybean meal for broilers
- *Hermetia illucens* was a second control, as it is considered a sustainable unconventional feedstuff
- Cardoon cake slightly affected the gut microbial profile and did not alter broiler performance in comparison to soybean meal or *H. Illucens*

## Introduction

The global demand for protein with a high nutritional value requires the development of efficient production systems that comply the three pillars of sustainability: environmental, economic and social sustainability (FAO 2021; FAO et al. 2021). Foods of animal origin provide humans with a good source of highly digestible protein, ensuring that the dietary requirement of essential amino acids is met. The poultry industry is characterised by its worldwide distribution, short production cycle and lower costs compared with other animal food production chains (FAO 2013), making it an ideal source of food products rich in protein. However, intensive farming systems are strongly criticised in terms of sustainability due to the significant problem of food *vs* feed competition and their extensive use of antibiotics (Makkar and Ankers 2014). Indeed, 80% of our ecological footprint can be traced back to feed production, thus the nutritional choices made for animals have a significant impact on the environment (Sala et al. 2017).

The dietary requirements of broilers change according to their developmental growth phase, necessitating specifically designed feeding strategies to meet the birds’ nutritional requirements in each growing phase as well as to minimise feeding costs (Moss et al. 2021). The content and the quality of dietary protein and energy sources in the feed are essential for optimal growth and well-being. Good feed quality in the first 10 days of life is fundamental for the correct development of the gut (e.g. intestinal villi) and its microbiota, which in turn favour maximal nutrient assumption in the adult bird. Feeding costs are greatest after 25 days of age due to the high feed intake (FI) of adult broilers (Neves et al. 2014). Soybean meal (SM) is an optimal ingredient for feeding to broilers. It has a high content of digestible protein, making it easy for feed formulations to meet the nutritional requirements of birds, but the environmental impact of soybean cultivation is high due to the extent of land required and deforestation practices employed to provide that land (van Hal et al. 2019).

Consumers are becoming increasingly aware of the subject of food sustainability and are thus paying greater attention to the ecological impact of their daily food choices. By consequence, companies have also begun to look at environmental sustainability programs with the prospect of developing entirely new business areas, and marketing to customers them accordingly (Gazzola et al. 2020).

Introducing even small changes to the poultry diet has the potential to bring about significant improvements in food production sustainability. One approach involves the improvement of feed efficiency, but this is a complex topic involving multifactorial matters such as genetics and precision feeding, thus identifying suitable solutions is not easy. However, replacing conventional ingredients based on soybean with unconventional ones, such as insect meals (IM) or by-products from the agro-industry, holds greater promise and could represent a significant turning point for the poultry industry.

In the last decade, several studies used unconventional feed ingredients, including insect meals, algae, single-cell proteins, and agro-industrial by-products (Carvalho et al. 2023; Pinotti et al. 2023). These ingredients have been investigated as alternatives to conventional protein sources in poultry nutrition. Agro-industrial by-products are generally inexpensive (https://www.agerborsamerci.it/listino-borsa/; Vastolo et al. 2022; Nunes et al. 2023) and readily available, making them attractive ingredients for animal feeding strategies in the case that their chemical and nutritional profiles are suitable for this purpose. This strategy also brings advantages linked to the valorisation of these biomasses, and producers also benefit from lower disposal costs.

Cardoon (*Cynara cardunculus* var. *altilis*) oil is used for human nutrition, as a bio-fuel and for the production of bioplastics (Fernández et al. 2006; Turco et al. 2019). Cardoon cake (CC) is the by-product of the oil extraction process. The exhausted residue, is a matrix rich in protein (approx. 30%), fibre (50%) omega-3 polyunsaturated fatty acids (PUFA) and bioactive compounds, such as polyphenols (PP) (Zumbo et al. 2022). Cardoon is considered a ‘tolerant species’ as it can be cultivated in the absence of irrigation, thereby presenting a good opportunity to reclaim and remediate marginal and unused lands, reducing the competition food vs feed for land and soil resources. Thus, CC is interesting from multiple perspectives: economic, nutritional and agronomic. However, little information is available on nutritional effects in poultry feeding strategy. The partial replacement of SM with CC would concur with more sustainable feeding plans for intensive poultry production. However, since CC has a high fibre content, there is the potential for a negative effect of its consumption on protein and energy absorption and, by consequence, on animal growth performances. Among the unconventional ingredients, black soldier fly (*Hermetia illucens*) meal has shown to be an effective protein source for partially replacing soybean meal in broiler diets (Bellezza Oddon et al. 2021). However, market fluctuations in the cost of IM have made it difficult for this novel ingredient to become a staple part of the broiler feeding strategy (Tavares et al. 2022). Despite the potential of CC as sustainable feedstuff for poultry, limited literature is currently available on this subject. Therefore, the present research aimed at testing the effects of a dietary replacement of SM with CC, at two substitution levels (15% and 20%), on the growth performances, gut microbial communities, carcase traits and the meat fatty acid profile in of broiler chickens. In addition, IM was included in this study as a second control to benchmark the nutritional and functional effects of CC against a well-documented but expensive alternative protein source.

## Materials and methods

### Experimental design

Animal handling was carried out in accordance with Italian Government guidelines (D.lgs 26/2014, protocol number 479/2020–PR). A total of 80 day-old Ross 308 male chicks were provided by a local hatchery (Incubatoio Settecrociari, Forlì - Cesena, Italy), where they were vaccinated against Marek’s disease, infectious bronchitis and Newcastle disease. Birds were randomly allotted into 4 dietary treatments composed of 5 replicated pens (4 animals per pen, individually identified by a ring). The sample size and the power analysis were computed using G*Power 3.1. The reference parameter for determining the minimum sample size is the feed conversion ratio, which takes into account both the daily weight gain and the daily feed intake. Based on scientific literature, the feed conversion ratio in broiler chickens ranges from 1 to 2, with a standard deviation of 0.1. The minimum sample size was determined assuming the need to detect a statistically significant difference between two means of at least one standard deviation, with a power of 0.9 and an alpha value of 0.05 (Snedecor and Cochran 1989). The experimental design was conceived in accordance with Bello et al. (2016), considering the pen as the experimental unit (five pens per dietary treatment).

Pens area was 1 m^2^ each and cocoa fibre was used as floor litter. Temperature range and set photoperiod were in accordance to Aviagen manuals (2018, 2022). The diets were formulated according to the National Research Council (1994) and adjusted to Aviagen manuals (2018, 2022) to meet animal requirements in each of three growth periods: starter (days 0–12), grower (days 13–21) and finisher (days 22–35). In this trial, CC was compared with two different protein sources used as references for their high level of nitrogen content: SM, the main conventional ingredient and IM, as an unconventional one. The inclusion level of CM was estimated based on previous results (Buccioni et al. 2020).

The feeding groups were: control group (C), fed the basal diet; HI15 group, fed the basal diet with 15% replacement of soybean meal (SM) with IM; CC15 group, fed the basal diet with 15% replacement of SM with CC; CC20 group, fed with the basal diet with 20% replacement of SM with CC. Animals were fed *ad libitum* and had free access to water. The diets were formulated to be isoproteic and isoenergetic (Table 1 and Table 2). The feeding trial lasted 35 days.

**Table 1.** Proximate profiles of each main protein source with main fatty acids.

Nutrients	SM^a^	IM^a^	CC^a^
CP^c^ g/100g on DM^b^	54.4	42.4	23.4
EE^d^ g/100g on DM	1.6	26.5	1.2
NDF^e^ g/100g on DM	10.2	10.8	59.3
ADL^f^ g/100g on DM	0.3	–	24.9
Ash	6.95	19.8	6.38
C12:0 g/100g of total FA^g^	0.1	34.76	0.01
C14:0 g/100g of total FA	0.2	14.5	0.01
C16:0 g/100g of total FA	11.2	18.6	10.9
C18:1cis9 g/100g of total FA	23.2	14.7	25.7
C18:2cis9cis12 g/100g of total FA	54.2	10.3	59.2
SFA^h^	1.53	3.62	2.9
PUFA^i^	2.47	1.12	0.48
n-3/n-6	0.13	0.23	0.01

^a^SM, soybean meal; IM, insect meal; CC, cardoon cake.

^b^DM, dry matter.

^c^CP, crude protein.

^d^EE, ether extract.

^e^NDF, neutral detergent fibre.

^f^ADL, acid detergent lignin.

^g^FA, fatty acids.

^h^SFA, saturated fatty acids.

^i^PUFA, polyunsaturated fatty acids.

**Table 2.** Ingredients and chemical composition of the experimental diets for broiler chickens in the different growing periods.

	C^a^			HI15^a^			CC15^a^			CC20^a^		
Ingredients and profile	starter	grower	finisher	starter	grower	finisher	starter	grower	finisher	starter	grower	finisher
Maize g/100g of DM^b^	64.25	67.45	67.65	64.25	67.45	67.65	64.25	67.45	67.65	64.00	67.45	67.00
Soybean meal g/100g of DM	21.50	21.00	20.00	19.00	17.85	18.50	17.27	16.85	16.00	16.20	16.00	16.00
*H. illucens* g/100g of DM	0.00	0.00	0.00	3.40	3.15	1.50	0.00	0.00	0.00	0.00	0.00	0.00
Cardoon cake g/100g of DM	0.00	0.00	0.00	0.00	0.00	0.00	3.23	3.15	3.00	4.00	4.00	3.7
Gluten g/100g of DM	2.00	0.50	0.00	1.60	0.50	0.00	3.00	1.5	0.00	3.55	1.50	0.00
Protein hydrolysates g/100g of DM	6.00	4.00	3.30	6.00	4.00	3.30	6.00	4.00	4.30	6.00	4.00	4.30
Sunflower oil g/100g of DM	2.00	2.83	5.00	1.50	2.83	5.00	2.00	2.83	5.00	2.00	2.83	4.95
CaHPO_4_ g/100g of DM	0.45	0.45	0.45	0.45	0.45	0.45	0.45	0.45	0.45	0.45	0.45	0.45
CaCO_3_ g/100g of DM	1.77	1.77	1.77	1.77	1.77	1.77	1.77	1.77	1.77	1.77	1.77	1.77
NaHCO_3_ g/100g of DM	0.23	0.23	0.23	0.23	0.23	0.23	0.23	0.23	0.23	0.23	0.23	0.23
NaCl g/100g of DM	0.23	0.23	0.23	0.23	0.23	0.23	0.23	0.23	0.23	0.23	0.23	0.23
DL Methionine g/100g of DM	0.14	0.14	0.14	0.14	0.14	0.14	0.14	0.14	0.14	0.14	0.14	0.14
Lysine g/100g of DM	0.38	0.35	0.18	0.38	0.35	0.18	0.38	0.35	0.18	0.38	0.35	0.18
Coline g/100g of DM	0.15	0.15	0.15	0.15	0.15	0.15	0.15	0.15	0.15	0.15	0.15	0.15
Premix^b^ g/100g of DM	0.91	0.91	0.91	0.91	0.91	0.91	0.91	0.91	0.91	0.91	0.91	0.91
DM^c^ g/100g of fresh feed	87.63	87.59	87.80	87.82	87.83	87.92	87.65	87.61	87.82	87.67	87.61	87.83
CP^d^ g/100g on DM	22.63	19.99	18.51	22.48	19.79	18.41	22.29	19.66	18.10	22.42	19.47	18.18
EE^e^ g/100g on DM	5.99	6.83	8.95	6.33	7.61	9.32	5.96	6.81	8.95	5.94	6.80	8.87
NDF^f^ g/100g on DM	3.18	3.24	3.18	3.23	3.24	3.18	4.80	4.82	4.68	5.17	5.26	5.07
Ca g/100g on DM	1.01	1.00	0.99	1.03	1.01	0.99	1.01	1.00	0.99	1.01	1.00	1.00
P g/100g on DM	0.52	0.48	0.46	0.52	0.48	0.46	0.51	0.48	0.47	0.51	0.48	0.48
Methionine g/100g on DM	0.54	0.48	0.46	0.54	0.48	0.46	0.52	0.47	0.45	0.52	0.46	0.45
Lysine g/100g on DM	1.39	1.26	1.06	1.42	1.26	1.06	1.27	1.14	0.98	1.24	1.11	0.98
Ash	2.45	2.42	2.35	2.91	2.80	2.53	2.38	2.36	2.26	2.36	2.36	2.30
ME^g^ Kcal/Kg DM	2,997	3,034	3,164	3,019	3,090	3,191	3,011	3,048	3,179	3,017	3,048	3,170
ME^g^ MJ/Kg DM	12.55	12.70	13.25	12.64	12.94	13.36	12.61	12.76	13.31	12.63	12.76	13.27

^a^C, Control (basal) diet; HI15, basal diet with 15% soybean meal substituted with insect meal; CC15, basal diet with 15% soybean meal substituted with cardoon cake; CC20, basal diet with 20% soybean meal substituted with cardoon cake.

^b^Vitamin and mineral premix.

^c^DM, dry matter.

^d^CP, crude protein.

^e^EE, ether extract.

^f^NDF, neutral detergent fibre.

^g^ME, metabolisable energy estimated according to Sauvant et al. (2002).

Animals from each pen were weighed individually on a weekly basis to calculate weight gain (WG). Weekly feed consumption was recorded for each pen as the difference between the offered and left over feed, and the individual feed intake (FI) was calculated by dividing the total amount of feed consumed by the number of animals in that pen. The feed conversion ratio (FCR) was calculated for each animal as the ratio of FI/WG.

Animal welfare was monitored during the whole period of the trial. Birds were checked daily, and any deaths recorded. To evaluate intestinal activity, each enclosure was divided into four equally sized virtual areas and scored from 1 to 3 (1 = used very little, 2 = average use, 3 = used a lot) according to its use for faeces deposition.

At the 35^th^ day, after 24 h of fasting all subjects were slaughtered (in an authorised slaughterhouse, Campi Bisenzio, Florence, Italy), plucked and eviscerated, and samples of gut digesta taken (from the caecum tract) from randomly selected animals for microbiological assay as described below. The carcases were then weighed for dressing out evaluation, and the meat was sampled for fatty acid (FA) characterisation as described below.

### Diet proximate analysis

Diet samples were collected at the start and end of each feeding period (starter, grower and finisher) according to ISO 24333:2009 procedure with the aim to obtain a representative sample (3 cumulative samples, one per each feeding period). Each cumulative sample was analysed for proximate profile to verify the correct supplies for animals. Crude protein (CP), ether extract (EE), crude fibre (CF), ash, Ca and P content were determined according to AOAC methods (976.06, 920.39, 962.09, 942.05, 985.33 and 984.27, respectively; AOAC 1995). Neutral detergent fibre (NDF) was determined according to van Soest et al. (1991), using heat stable amylase and sodium sulphite, and expressed inclusive of residual ash.

Diet metabolisable energy (ME) was estimated from feed tables according to Sauvant et al. (2002).

The chemical and nutritional profile of the basal diet are reported in Table 2.

The same procedure was used to determine the main proximate characteristics of the raw ingredients (Table 1). The fatty acid profile was determined according to Sukija and Palmquist (1990).

### Carcase traits

Dressing out percentages were computed after slaughter as the ratio between the eviscerated warm carcase weight and the live weight of each bird. Then, carcases were chilled at 4 °C and transported to University laboratories for analysis by 24h from slaughtering. Based on consumer preferences and choices, selected carcase traits (legs, fat, breast, liver, gizzard) from each carcase (80 subjects) were weighed and recorded (Barbut 2017).

### Fatty acid profile of meat

Fat was extracted from samples of homogenised breast meat (2.5 g) from each subject, according to Folch et al. (1957). The lipid extract (50 mg) was suspended in n-Hexane solution (2 mL) containing C19:0 methyl ester as internal standard to obtain a fat concentration of approximately 25 mg/mL.

The FA profile was determined following the basic transmethylation of glycerolipids, according to Christie (1982), and fatty acid methyl esters (FAME) were detected by gas-chromatographic analysis, according to Scicutella et al. (2021). Data are expressed as g/100 g of lipid extract.

### Characterisation of the caecum microbial community

Samples of caecum content were collected in the immediate post-mortem from four birds per treatment, selected at random in the slaughterhouse. Samples were transferred to 50 mL vials and stored at −80 °C until DNA extraction. To avoid variation in the cutting procedure, the same operator was employed for all animals.

### DNA extraction, amplicon preparation and sequencing

Samples of caecum content were thawed, and 250 mg of each caecum content was used for DNA extraction using the Fast DNA Spin kit for soil (MP Biomedicals, Solon, OH), as previously reported by Daghio et al. (2021). It was not possible to extract the DNA from one of the samples collected from the control group since the caecum was damaged.

The V3-V4 region of the 16S rRNA gene was amplified with Pro341F and Pro805R primers (Takahashi et al. 2014). Sequencing was performed at IGA Technology Services s.r.l. (Udine, Italy) by MiSeq Illumina (Illumina, Inc., San Diego, CA, USA) using a 300 bp x 2 paired-end protocol. Sequencing produced a total of 1,429,343 reads with an average of 95,290 ± 5,810 reads per sample (average ± standard error).

### Bioinformatics

Bioinformatic elaborations were performed in R 4.2.1 (R Core Team 2020) with the DADA2 package (Callahan et al. 2016), version 1.24.0. Primer sequences were removed using Cutadapt (Martin 2011). Forward reads were truncated at 260 bases and reverse reads were truncated at 250 bases. Reads with expected errors greater than 2 were discarded. Specific error rates were estimated for both the forward and reverse reads and used to infer the amplicon sequence variants (ASVs) on the dereplicated reads. The read pairs were merged with default parameters and chimeric sequences were removed. Taxonomic assignment for each ASV was performed against the SILVA 138.1 database (Pruesse et al. 2007) (confidence 80%). The ASVs with a relative abundance lower than 0.01% in all samples were removed from the dataset. A total of 662,662 high-quality sequences were obtained with an average of 44,177 ± 3,429 sequences per sample (average ± standard error).

### Statistical analysis

Data related to the weekly WG and FCRor to each feeding period (starter, grower and finisher) were processed according to a completely randomised design with repeated measures using the SAS MIXED model in which diet (D) and time (T) were the fixed effects (SAS 2008). The pen was considered the experimental unit, and it was included in the model as a random effect nested within the main treatment (D):

$$Y_{\text{ijkl}}=\text{ μ }+D_{i}+T_{j}+I_{k}(D_{i})+D_{i}\text{ X }T_{j}+\varepsilon_{\text{ijkl}}$$

where Y_ijkl_ = variable; µ = overall mean; D_i_ = fixed effect of the i^th^ diet (1 to 4); T_j_ = fixed effect of the j^th^ measuring time (1 to 5); I_k_ = random effect of the k^th^ pen nested within the diet (1 to 5); and ε_ijkl_ = residual error. The covariance structure had a compound symmetry, which was selected based on the Akaike’s information criterion of the SAS MIXED model (2008). The statistical significance of the effect of the dietary treatment was tested against the variance of the bird nested within the diet, according to the repeated measures design theory (Littell et al. 1998). Multiple comparisons between means were performed using Tukey’s test.

Data related to the WG, FCR of the whole period, physical parameters of the meat, dressing out, carcase traits and meat sample fatty acid profiles were analysed using the following linear model (SAS 2008):

$$Y_{\text{ikl}}=\text{ μ }+D_{i}+I_{k}(D_{i})+\varepsilon_{\text{ikl}}$$

where Y_ijkl_ = variable; µ = overall mean; D_i_= fixed effect of the i^th^ diet (1 to 4); I_k_= random effect of the k^th^ pen (1 to 5), and ε_ijkl_= residual error. Multiple comparisons among means were performed using Tukey’s test.

The probability of a significant effect due to experimental factors was fixed for p-value < 0.05.

Univariate analysis of bacterial genera was performed using the following linear model (JMP 17Pro software; SAS Institute Inc., Cary, NC):

$$Y_{i}=\text{ μ }+D_{i}+e_{i}$$

where Y_i_ = bacterial genera; µ= overall mean; D_i_ = fixed effect of the i^th^ diet (1 to 4); ε_i_= random error.

Multivariate analysis of bacterial genera was performed using three techniques to discriminate the four groups: canonical discriminant analysis (CDA), stepwise discriminant analysis (SDA) and discriminant analysis (DA) as previously described by Conte et al. (2018). Both the univariate analyses and the multivariate analyses were performed on transformed genera abundances (log_2_(*x* + 1)). The CDA is a dimension reduction technique able to perform both univariate and multivariate one-way analyses. CDA derives a set of new variables, called canonical functions (CAN), as a linear combination of the original interval variables, as shown in the following equation:

$$\text{CAN}=d_{1}X_{1}+d_{2}X_{2}+\ldots +d_{n}X_{n},$$

where d_i_ = canonical coefficients that designate the involvement of each variable in composing the CAN, and X = scores of the n original variables.

In general, if a total of k groups are involved in the study, then *k* − 1 CAN are extracted. The effective separation between groups was assessed using the Mahalanobis distance and the corresponding Hotelling’s t-square test (De Maesschalck et al. 2000).

The minimum number of bacterial genera able to discriminate the four groups was ascertained by SDA, a statistical technique specifically conceived to select the subset of variables best able to separate groups.

The ability of CAN to assign each animal to the four groups was calculated as the percentage of correct assignments using DA (Mardia et al. 2000). In practice, the CAN was applied to each animal, thus obtaining a value called the discriminant score. The centroid for each group was then calculated and the distances between each individual and the four centroids evaluated.

## Results

### Animal welfare

The space provided by the pens allowed the birds’ physiological requirements to be met and the ethological characteristics of the specie to be expressed (Scientific Committee on Animal Health and Animal Welfare 2000). Over the entire trial period, all subjects were always active, but birds fed CC15 and CC20 showed more reactivity than those receiving the C or HI15 diets. No direct trauma, lameness or mutilation lesions were observed, and no symptoms of respiratory or enteric diseases or deaths occurred. The cloacal area and hocks remained clean. All pathogen assays and parasitological examinations gave negative results. All four groups used the entire pen area for faeces deposition in a uniform manner, with an average score of 1 (used very little). The faeces from animals fed CC15 and CC20 were more compact compared with those from the animals fed C and HI15.

### Animal performances, carcase traits and meat fatty acid composition

During the starter period, chicks fed CC15 showed a similar WG, FI and FCR to those fed C (p-value = 0.001, p-value < 0.001, p-value = 0.030, respectively), and a lower FI than the HI15 and CC20 groups (p-value < 0.001), as reported in Table 3. WG in the animals fed CC20 was similar to that for the other groups, and animals in CC20 also consumed a similar quantity of feed to those fed HI15, but FCR was greater than in the latter group. The FCR of HI15 was comparable to that of C and CC15. In the first week of life, the performances (WG, FI and FCR) of birds fed CC15 were the same of those fed HI15 (p-value = 0.001, p-value < 0.001, p-value = 0.003, respectively). This trend was not maintained in the second week of life when only WG performance in CC20 was comparable to that in C and HI15, whereas differences were seen in the other parameters (Table 3; p-value < 0.001, p-value < 0.001, p-value = 0.013 for WG, FI and FCR, respectively).

**Table 3.** Live performance of broiler chickens fed the experimental diets (C, HI15, CC15 and CC20) based on the feeding period (starter, grower, finisher, whole period) and single week of the experiment.

Feeding period	Item^c^	C^a^	HI15^a^	CC15^a^	CC20^a^	SEM^b^	p-value
Starter	WG (kg/bird)	0.383^ab^	0.439^a^	0.349^b^	0.382^ab^	0.015	0.001
	FI (kg/bird)	0.736^bc^	0.775^ab^	0.691^c^	0.813^a^	0.014	<0.001
	FCR	1.951^ab^	1.814^b^	2.052^ab^	2.223^a^	0.096	0.030
Grower	WG (kg/bird)	0.810^b^	0.966^a^	0.861^ab^	0.799^b^	0.037	0.010
	FI (kg/bird)	1.179^bc^	1.298^a^	1.144^c^	1.203^b^	0.015	<0.001
	FCR	1.519	1.395	1.393	1.558	0.075	0.302
Finisher	WG (kg/bird)	0.811	0.800	0.703	0.688	0.041	0.086
	FI (kg/bird)	1.843^b^	2.238^a^	1.768^b^	1.857^b^	0.046	<0.001
	FCR	2.359^b^	2.901^a^	2.694^a^	2.941^a^	0.159	0.050
Whole period	WG (kg/bird)	2.032^ab^	2.204^a^	1.916^ab^	1.850^b^	0.080	0.016
	FI (kg/bird)	2.994^b^	3.522^a^	2.958^b^	3.077^b^	0.042	<0.001
	FCR	1.760	1.886	1.840	1.946	0.074	0.394
1st week	WG (kg/bird)	0.101^b^	0.128^a^	0.122^a^	0.114^ab^	0.005	0.001
	FI (kg/bird)	0.174^b^	0.178^b^	0.167^b^	0.207^a^	0.006	<0.001
	FCR	1.788^ab^	1.455b^c^	1.393^c^	1.905^a^	0.101	0.003
2nd week	WG (kg/bird)	0.286^a^	0.311^a^	0.233^b^	0.269^ab^	0.011	<0.001
	FI (kg/bird)	0.549^b^	0.597^a^	0.521^b^	0.606^a^	0.008	<0.001
	FCR	1.976^b^	1.987^ab^	2.300^ab^	2.381^a^	0.100	0.013
3rd week	WG (kg/bird)	0.398^ab^	0.449^a^	0.383^b^	0.372^b^	0.016	0.009
	FI (kg/bird)	0.670^ab^	0.700^a^	0.622^bc^	0.597^c^	0.012	<0.001
	FCR	1.721	1.646	1.682	1.656	0.084	0.928
4th week	WG (kg/bird)	0.461	0.517	0.493	0.427	0.023	0.272
	FI (kg/bird)	0.834^b^	0.950^a^	0.794^b^	0.759^b^	0.025	<0.001
	FCR	1.881	1.942	1.708	1.865	0.103	0.497
5th week	WG (kg/bird)	0.536	0.489	0.431	0.442	0.031	0.699
	FI (kg/bird)	0.896^b^	1.097^a^	0.860^b^	0.908^b^	0.024	<0.001
	FCR	1.875	2.302	2.104	2.169	0.120	0.122

^a^C, Control (basal) diet; HI15, basal diet with 15% soybean meal substituted with insect meal; CC15, basal diet with 15% soybean meal substituted with cardoon cake; CC20, basal diet with 20% soybean meal substituted with cardoon cake.

^b^SEM, standard error of the mean.

^c^WG, weight gain; FI, feed intake; FCR, feed conversion ratio.

Means within a row lacking a common superscript differ (p-value < 0.05).

In the grower period, no significant differences were found in FCR between groups (p-value = 0.302). The WG of birds fed CC15 was similar to that of the other groups, while FI was lower than that reported for HI15 and CC20 (p-value < 0.001). Instead, the FI of animals in CC20 was comparable to that for those in C, but lower than those in HI15 (Table 3; p-value < 0.001). In the third (p-value = 0.009, *p* < 0.001, p-value = 0.928 for WG, FI and FCR, respectively) and fourth (p-value = 0.272, p-value < 0.001, p-value = 0.497 for WG, FI and FCR, respectively) weeks of life, birds fed the diets integrated with CC showed similar performances (regardless of the inclusion level), but no differences were found between groups in FCR (Table 3; p-value = 0.928, p-value = 0.497 for the third and fourth week, respectively).

In the finisher period, WG in the animals of CC15 and CC20 did not differ from C and HI15 (p-value = 0.086), but the FCR worsened in both groups fed CC with respect to C, whilst remaining comparable to that of HI15 (Table 3; p-value = 0.050). FI was greatest in the animals fed HI15 (p-value < 0.001).

In the fifth week of life, FI was greater in the animals fed HI15 than in all other groups (p-value < 0.001). Considering the whole period, birds fed CC showed similar WG and FI with respect to C (p-value = 0.016, p-value < 0.001 respectively), but no differences were found in FCR (Table 3; p-value = 0.394).

Finally, the dressing out values were similar for all groups: C, 76.33%; HI15, 78.54%; CC15,77.02%; CC20, 76.74%; SEM 1.15 (p-value = 0.318).

The main carcase traits were also similar between groups. No significant differences were found in breast, thigh and liver weights or in the weight percentage of breast and thighs (Table 4).

**Table 4.** Carcase traits of broiler chickens fed with diets C, HI15, CC15 and CC20.

Edible part	C^a^	HI15^a^	CC15^a^	CC20^a^	SEM^b^	p-value
Whole carcase, kg	1.800	2.018	1.880	1.758	0.108	0.561
Breast, g	391.25	428.85	383.75	375.00	39.73	0.625
Breast, g/100 g of edible parts	21.61	21.18	20.42	21.34	1.21	0.533
Thigh, g	442.50	485.00	485.00	433.75	31.66	0.698
Thigh, g/100 g of edible parts	24.73	24.03	25.64	24.66	0.61	0.325
Liver, g	46.25	45.00	43.75	42.50	3.54	0.970
Liver, g/100 g of edible parts	2.58	2.24	2.32	2.41	0.15	0.423

^a^C, Control (basal) diet; HI15, basal diet with 15% soybean meal substituted with insect meal; CC15, basal diet with 15% soybean meal substituted with cardoon cake; CC20, basal diet with 20% soybean meal substituted with cardoon cake.

^b^SEM, standard error of the mean.

The FA profile of breast meat from animals fed the diets containing CC was richer in oleic acid (C18:1 cis9, OA, p-value = 0.035) and lower in linoleic acid (C18:2 cis9 cis12; LA, p-value < 0.001) than those fed C or HI15. The presence of IM in the diet increased the C12:0 and C14:0 concentrations (p-value < 0.001, p-value = 0.001, respectively), while the content of α-linolenic acids was lower in animals fed CC20 (C18:3 cis9 cis12 cis15, αLNA, p-value = 0.022). No difference in C16:0 or C18:0 was found between the groups (Table 5; p-value = 0.669, p-value = 0.876, respectively).

**Table 5.** Fatty acid content of breast meat from chickens fed with diets C, HI15, CC15 and CC20.

Fatty acid (g /100 g of fatty acids)	C^a^	HI15^a^	CC15^a^	CC20^a^	SEM^b^	p-value
C12:0	0.342^b^	0.761^a^	0.248^cb^	0.180^c^	0.059	<0.001
C14:0	0.499^c^	0.831^a^	0.596^b^	0.441^c^	0.050	0.001
C16:0	18.684	17.864	19.443	18.580	0.885	0.669
C16:1 cis9	3.005	3.492	3.492	3.169	0.455	0.835
C18:0	7.358	6.768	6.844	7.068	0.557	0.876
C18:1 cis9	38.739^c^	39.234^c^	40.445^b^	42.202^a^	0.509	0.035
C18:2 cis9 cis12	24.731^a^	23.092^b^	20.527^c^	19.850^c^	0.789	<0.001
C18:3 cis9 cis12 cis15	0.338^a^	0.291^ab^	0.286^ab^	0.242^b^	0.039	0.022
C20:3 n6 cis8 cis11 cis14	0.457	0.401	0.424	0.461	0.055	0.849
C20:4 cis5 cis8 cis11 cis14	2.872	2.531	2.192	2.318	0.437	0.713
SFA	29.932	28.315	29.500	28.004	1.337	0.7033
PUFA	28.842^a^	26.731^a^	23.893^b^	23.508^b^	0.9010	0.0037
n-3/n-6	0.019	0.0180	0.020	0.019	0.002	0.8914

^a^C, Control (basal) diet; HI15, basal diet with 15% soybean meal substituted with insect meal; CC15, basal diet with 15% soybean meal substituted with cardoon cake; CC20, basal diet with 20% soybean meal substituted with cardoon cake.

^b^SEM, standard error of the mean.

Means within a row lacking a common superscript differ (p-value < 0.05).

### Microbial characterisation

**Figure 1.**
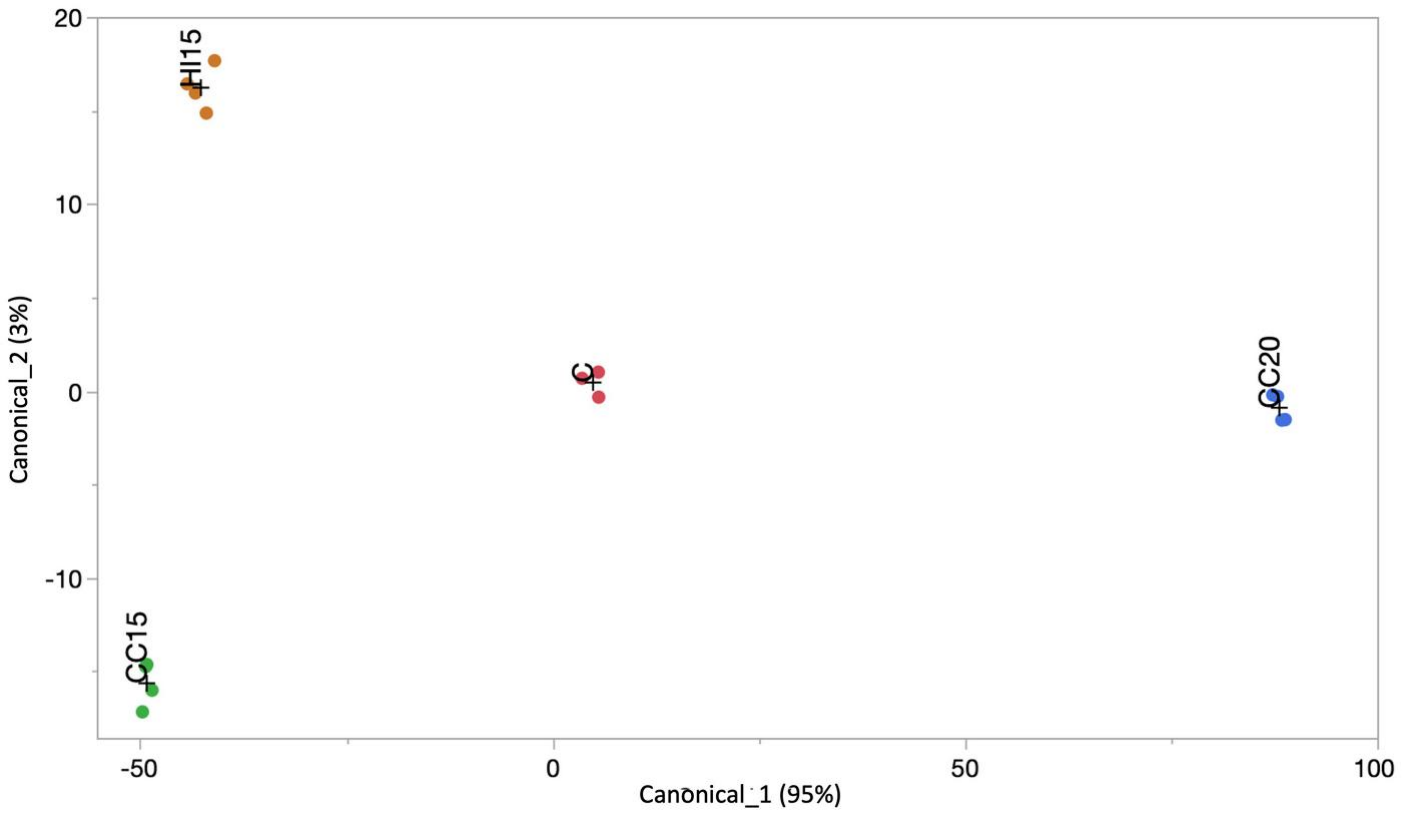
Results of the canonical discriminant analysis. The tested diets changed the composition of the gut microbial communities.

The partial replacement of SM with CC did not affect the microbial profile of the gut at either inclusion level. Nevertheless, several interesting differences did come to light (Table 6 and Figure 1). *Bifidobacterium* was detected in the CC20 group only, which was also the group with significantly lower abundance of *Bacteroides* (p-value = 0.031), *Monoglobus* (p-value = 0.044), *UCG-009* (p-value < 0.001), *Colidextribacter* (p-value = 0.011) and *NK4A214_group* (p-value = 0.001) with respect to the other three groups. *Pseudomonas* (p-value = 0.001) and *Latilactobacillus* (p-value = 0.014) showed the highest abundances in CC20, whereas they were at their lowest in HI15. The abundance of *Sellilomonas* was similar in C with respect to the CC15 group, and similar in HI15 with respect to CC20, but the inclusion of IM or CC at 20% had a negative effect on the abundance of this genus (p-value = 0.008). *Flavonifractor*, whilst present C, CC15 and CC20, was not detected in HI15; the highest value for this genus was detected in CC15 (p-value < 0.001).

**Table 6.** Taxonomic composition of the microbial communities at genus level expressed as log_2_(*x* + 1) of genera abundances.

Genus	C^a^	HI15^a^	CC15^a^	CC20^a^	SEM^b^	p-value
*Bifidobacterium*	0.000^b^	0.000^b^	0.000^b^	3.972^a^	0.107	<0.001
*Corynebacterium*	5.045	5.191	5.797	5.319	0.560	0.815
*Bacteroides*	12.691^a^	12.147^a^	12.557^a^	11.150^b^	0.326	0.031
*Barnesiella*	9.688	9.994	9.984	7.915	1.250	0.610
*Alistipes*	11.276	10.702	10.122	9.592	0.493	0.186
*Rikenella*	11.741	10.212	9.82	8.804	1.060	0.383
*Helicobacter*	7.060	8.258	6.768	6.356	1.285	0.752
*Erysipelatoclostridium*	6.240	6.162	5.459	4.651	0.468	0.129
*Erysipelotrichaceae_UCG-003*	4.697	4.861	5.528	4.505	0.363	0.275
*Carnobacterium*	7.245^a^	0.000^c^	4.986^b^	8.089^a^	0.742	<0.001
*Enterococcus*	11.367	10.408	11.218	11.332	0.484	0.497
*Lactobacillus*	8.806	7.604	8.948	8.207	1.568	0.929
*Latilactobacillus*	8.566^b^	5.480^c^	8.048^b^	11.919^a^	1.113	0.014
*Ligilactobacillus*	6.265	7.229	6.735	7.654	0.804	0.695
*Streptococcus*	9.793	10.972	9.460	10.987	1.264	0.769
*Staphylococcus*	6.379	5.385	6.515	5.255	0.734	0.541
*Christensenellaceae_R-7_group*	8.604	8.772	9.668	8.645	0.490	0.424
*[Ruminococcus]_torques_group*	10.192	10.019	10.952	8.864	0.505	0.082
*Blautia*	8.431	8.683	9.800	7.953	0.431	0.064
*Frisingicoccus*	4.985	4.495	4.920	4.672	0.485	0.894
*Lachnoclostridium*	6.038	6.541	6.796	5.023	0.438	0.066
*Lachnospiraceae_UCG-010*	6.603	6.440	6.480	5.697	0.519	0.628
*Sellimonas*	6.414^a^	5.182^b^	6.613^a^	5.348^b^	0.281	0.008
*Shuttleworthia*	5.490	5.747	6.391	4.674	0.439	0.104
*Tuzzerella*	6.293	6.808	6.844	6.829	0.576	0.913
*Monoglobus*	8.607^a^	8.025^a^	8.742^a^	7.177^b^	0.360	0.044
*UCG-008*	5.961^a^	4.278^b^	0.000^c^	4.524^b^	0.225	<0.001
*UCG-009*	4.249^b^	5.502^a^	5.593^a^	3.348^c^	0.274	<0.001
*Colidextribacter*	6.691^a^	6.643^a^	7.128^a^	4.851^b^	0.411	0.011
*Flavonifractor*	5.100^b^	0.000^c^	5.902^a^	4.515^b^	0.311	<0.001
*NK4A214_group*	8.197^a^	8.087^a^	8.723^a^	5.762^b^	0.389	0.001
*Oscillibacter*	5.488	5.205	6.112	5.798	0.619	0.761
*Oscillospira*	6.306	5.773	6.020	5.513	0.582	0.827
*UCG-005*	10.675	8.991	10.949	8.996	0.665	0.123
*DTU089*	4.086	5.834	5.374	4.727	0.722	0.441
*Faecalibacterium*	8.694	7.653	9.198	7.768	0.638	0.341
*Ruminococcaceae Incertae_Sedis*	6.679	7.542	8.173	6.791	0.527	0.237
*Negativibacillus*	4.273	3.826	4.625	4.170	0.353	0.488
*Paludicola*	6.179	4.613	5.581	6.607	0.990	0.547
*Ruminococcus*	7.508	7.619	6.271	7.001	0.487	0.262
*Subdoligranulum*	7.795	9.583	9.075	9.495	0.705	0.384
*[Eubacterium]_brachy_group*	4.330	3.169	4.911	4.138	0.787	0.500
*Family_XIII_AD3011_group*	4.999	5.293	5.712	5.080	0.430	0.681
*Romboutsia*	6.609	7.055	7.379	6.045	1.232	0.881
*Pseudomonas*	7.906^b^	4.062^c^	8.199^b^	10.621^a^	0.806	0.001
*Akkermansia*	11.624	11.415	9.478	10.046	0.581	0.079
Unknown	13.316^b^	13.476^b^	14.042^a^	12.214*^c^*	0.270	0.004

^a^C, Control (basal) diet; HI15, basal diet with 15% soybean meal substituted with insect meal; CC15, basal diet with 15% soybean meal substituted with cardoon cake; CC20, basal diet with 20% soybean meal substituted with cardoon cake.

^b^SEM, standard error of the mean.

Means within a row lacking a common superscript differ (p-value < 0.05).

From the 47 genera detected in the caecum, the following seven were retained (p-value < 0.001) at the end of the stepwise discriminant analysis: *Bifidobacterium*, *Enterococcus*, *Ligilactobacillus*, *[Ruminococcus]_torques_group*, *UCG-008*, *Flavonifractor*, and *Ruminococcaceae Incertae_Sedis*. The selected variables had a high discriminant power, with R^2^ ranging from 0.40 for *Ruminococcaceae Incertae_Sedis* to 0.67 for *Bifidobacterium*, *Ligilactobacillus* and *Flavonifractor* (data not shown). Some of the selected variables (*Enterococcus*, *Ligilactobacillus*, *[Ruminococcus]_torques_group* and *Ruminococcaceae Incertae_Sedis*) did not differ between groups according to the ANOVA model used; this was a consequence of the multivariate approach which evaluates the relationship between variables considering their correlation matrix.

## Discussion

This study investigated the effects of CC as an unconventional ingredient in a poultry feeding strategy. We compared animal performances and the intestinal microbial profile of animals receiving a diet in which part of the feed’s protein source came from CC compared with those of animals fed either a conventional diet containing SM (the conventional protein source) or a diet containing IM, the unconventional protein source ‘par excellence’. The evaluation considered the whole growing period for broilers (35 days; Table 3). Despite its higher fibre content, the inclusion of CC in the diet at the substitution levels of 15% or 20% had no negative effect upon animal performance in terms of FCR compared with the diets based on conventional or another novel protein source (SM and IM, respectively), thus challenging the notion that ingredients high in fibre have a detrimental impact on performance. Indeed, whilst monogastrics have difficulty in digesting fibre, it is nevertheless important for gut health as it favours intestinal motility and the production of short-chain fatty acids during its fermentation. The starter period was confirmed to be crucial for chicks, with the second week of life shown to be the most critical in the present trial. From the age of 7 to 14 days old, animals fed CC20 showed the worst FCR, although WG remained comparable to that of birds fed the C or HI15 diets. In the starter period (days 1–12), the growth of animals fed CC15 was comparable to that of C, while that of animals belonging to CC20 was characterised by the worst performance in terms a low FCR with a high FI. This suggests that the inclusion level of CC in the diet should be less than 20 g/100 g of DM, and that the chicks fed the CC20 diet compensated for the lower protein quality and higher fibre content of CC by increasing their FI to ensure that their nutritional requirements were met. Ross broilers are particularly sensitive to amino acid levels in the diet and to the digestibility of feed protein, responding well to properly balanced diets, as recommended in the Ross-Broiler handbook nutrition specifications (2018). A high level of digestible amino acids was shown to be fundamental for good animal performance and welfare. Considering that the majority of feeding costs are encountered after 25 days of age due to the higher daily FI of adult broilers with respect to chicks, the 14% decrease in FCR in the early phases of life is largely compensated for by the lower cost of CC with respect to SM over the whole rearing period. Considering the FCR over the entire rearing period, all groups were comparable, since no significant differences were observed in WG or FI for the animals fed C, CC15 or CC20. This was confirmed by the similar dressing out values and the carcase traits of edible parts between groups. The inclusion of CC in the feed improved the nutritional quality of meat by increasing the OA content, a functional FA considered beneficial for human health by EFSA (2011). As poultry meat is recognised to be a good source of noble protein for human nutrition, an improvement in the quality of the meat’s lipid fraction is certainly of added value.

More than 600 different species of bacteria have been identified in the chicken gut (Torok et al. 2011), and the important role of the microbiota for host health and productivity is well defined (Ducatelle et al. 2023). The colonisation of the gut by microbial species occurs immediately after hatching and it is strongly affected by environmental conditions and the feeding strategies adopted by the farm. The gut microbiota plays an important role in nutrient utilisation and absorption, the fermentation of fibre for volatile fatty acid production, and the synthesis of certain vitamins, all of which strongly affect the well-being of chickens. Previous studies have investigated the relationship between the composition of gut microbiota and animal performances in broiler chickens. Taxa such as *Oscillibacter*, *Butyricicoccus* and members of the family *Enterobacteriaceae* were more abundant in high-efficiency animals, microorganisms such as *Acinetobacter*, *Bacteroides*, *Lactobacilus*, *Subdoligranulum* and related *Peptostreptococcaceae* members were negatively associated with feed efficiency (Liu et al. 2021; Dittoe et al. 2022). In our work, the inclusion of CC in the diet did not compromise animal performances over the whole growing period. Microorganisms belonging to the genera *Lactobacilus*, *Oscillibacter* and *Romboutsia* (family *Peptostreptococcaceae*) were detected in the caecum microbiome in this trial (Table 6), but their relative abundance did not change between the diets.

Multivariate analysis confirmed the diet to be a key factor affecting the intestinal microbiota, even if the differences in the microbial community composition were found to be minimal between the different diets studied. The selected bacterial genera were able to discriminate the four feeding groups. Both diets supplemented with CC were distant from C and HI15. Cardoon is rich in fibre, which favours the intestinal production of butyric acid and polyphenols, known antimicrobials. Hence, we can also hypothesise that CC has an effect on the microbiota profile. Indeed, the genus *Bifidobacterium* was only detected in the gut of animals fed the diet containing 20 g CC per 100 g DM to replace 20% of SM. This was probably due to the higher dietary fibre content of the CC20 diet compared with the other three since *Bifidobacterium* is very efficient at degrading fibre, with an energy conversion rate of 1 mol glucose into 2.5 mol of ATP with respect to the *Lactobacilli* that produce only 2.0 mol of ATP (Palframan et al. 2003). Recently, Wang et al. (2022) highlighted the important role of *Bifidobacteria* in promoting intestinal health, suggesting the combined use of dietary fibre plus these microorganisms in nutritional strategies in humans. Through fibre fermentation, these microorganisms produce monosaccharides for their growth. Hence, fibre quality is an important factor affecting the presence of *Bifidobacteria* in the gut (Wang et al. 2022). The degree to which fibre is polymerised impacts its utilisation by *Bifidobacterium* by influencing the microorganisms’ ability to ferment the substrate, which is nonetheless low. The presence of *Bifidobacteria* in the microbiota of only the chickens fed CC20 suggests that the quality of the fibre in CC and the quantity provided by the CC20 diet provided the optimal conditions for both the proliferation of fibrolytic bacteria and the biological activity of these microorganisms in the gut. *Bifidobacteria* produce short-chain fatty acids, including acetic, propionic and butyric acids, the antimicrobial action of which is well-reported in the literature (van Immerseel et al. 2005; Antongiovanni et al. 2007, 2010; Scicutella et al. 2021).

The results of this study showed the *Bacteroides* genus to be affected by diet, being negatively affected by the replacement 20% SM with 20 g/100 g on DM of CC. This microbial genus is composed of saccharolytic bacteria that are also capable of proteolytic fermentation (Cummings and Macfarlane 1991). In several *in vivo* and *in vitro* trials, *Bifidobacterium* and *Bacteroides* showed different behaviours against fermentable carbohydrates in function not only of the strain but also of the kind of oligosaccharides present in the medium, showing a symbiotic activity. In particular, *Bacteroides* degrade inulin-type fructans, whilst *Bifidobacteria* do not, which instead use metabolites of inulin fermentation. Cardoon contains inulin, but it is mainly present in the roots and buds (Melilli et al. 2020), whereas polyphenols and cardosins are concentrated in leaves and flowers, respectively (Christaki et al. 2012). Because CC is rich in polyphenols, which exert antimicrobial activities in a selective manner, and because it is probably poor in inulin, the increase in this ingredient in the diet from the 15% to the 20% inclusion level did not generate favourable condition for the growth of *Bacteroides*.

Cardoon cake inclusion in the broiler diet enhanced the proliferation of *Latilactobacillus*, a carbohydrate utiliser, also considered to be a probiotic. This bacterium concurs in regulating gut microbiota, acting as an anti-inflammatory agent through the promotion of IL-10 production. It also has several genes encoding for bacteriocins, which inhibit the growth of a large variety of pathogens (Chen et al. 2020). *Latilactobacillus* appeared to be sensitive to the dietary inclusion of black soldier fly IM, probably due to the antimicrobial activity of chitosan and lauric acid contained in this ingredient. *Carnobacterium* is a ubiquitous lactic acid bacterium, and although it can catabolize a range of carbohydrates, including chitin (Coombs and Brenchley 1999), it is inhibited by acetate (Whitman 2015). The abundance of this genus was lower in the guts of animals fed the HI15 diet, whereas it was greater in CC20 with respect to HI15 and CC15. A possible hypothesis to explain this finding could be formulated on the ability of *Carnobacterium* to use arginine as an additional energy source when environmental conditions are not favourable for its growth or survival (Marquis et al. 1987).

In our study, the abundance of *Colidextribacter* was significantly lower in animals fed the higher substitution level of CC (CC20). The literature reports that the proliferation of this genus is favoured by diets containing a high level of fat, whereas it is penalised by the presence of polyphenols or inulin (Wu et al. 2022). Hence, it is feasible that it was the presence of these functional molecules in CC that inhibited the growth or the survival of *Colidextribacter*, but only when they attained a certain concentration in the diet (Wu et al. 2022).

*Flavonifractor* is a flavonoid-degrading bacterium capable of alleviating the inflammatory responses of its host’s adipose tissue. Our findings showed no or only a small positive effect of CC in the diet upon the abundances of this genus, again showing that the use of this unconventional ingredient has the potential to exert beneficial effects upon broiler health.

## Conclusions

The world population is increasing. To ensure survival and social well-being, steps must be taken to safeguard the capacity of food chains to meet the nutritional needs of people worldwide. Meat is rich in easily digestible noble protein, an important source of essential amino acids, fundamental for good health in children and the elderly especially. Poultry production offers a feasible way of satisfying these nutritional requirements as the production cycle is short and less expensive than that of larger animals. In addition, the sustainability of animal production chains must improve to bring about greater efficiency in the use of resources, and the use of by-products from other agro-industry productions in the feeding strategy is one of the ways through which this can be achieved. Cardoon cake, at the inclusion levels adopted in this trial, was well tolerated by Ross 308 broilers despite its high fibre content and the presence of polyphenols. It did not compromise animal performances over the whole growing period and it enhanced the proliferation of two probiotic genera in the gut, namely *Latilactobacillus* and *Bifidobacteria*, suggesting that the quality of fibre in CC contributed to providing the optimal conditions for both the proliferation of fibrolytic bacteria and the biological activity of these microorganisms in the gut.

## Data Availability

The data that support the findings of this study are available from the corresponding author, MD, upon reasonable request. Sequencing data are available at the National Centre for Biotechnology Information (NCBI), BioProject number PRJNA1221821.
